# Supplementary material for: Antinociceptive and anti-inflammatory effects of hydrazone derivatives and their possible mechanism of action in mice
Source: PLoS One. 2021 Nov 24;16(11):e0258094. doi: 10.1371/journal.pone.0258094 (PMC8612535; doi:10.1371/journal.pone.0258094)
Supplement: S1 File — (DOCX) [file pone.0258094.s001.docx]

**Attachment**

**Preparation of the intermediate carbonyl compounds**

In a reaction flask, 2mmol of each aldehyde (a1-a5), 10 ml of propanone PA (higher excess) and 10 ml of an aqueous solution of KOH at 56 g/l were mixed. The reaction mixture was closed and left to react at room temperature for 24 h (protected from light). The resulting mixture was poured into a beaker containing 50 ml of a solution of H_3_CCOOH 0.20 mol/l and left in refrigerator for another 24 h for crystallization. The solid formed was filtered, washed with distilled water and dried at room temperature (protected from light). These synthetic intermediates (i1-i5) were characterized only by melting point determination since their syntheses and properties are already reported in scientific papers.

**Intermediate properties**

*Intermediate* i1: 4-(2-(hydroxyphenyl)but-3-en-2-one

yellow solid, 65% yield; mp 134-136 ^o^C.

*Intermediate* i2: 4-(4-(hydroxyphenyl)but-3-en-2-one

yellow solid, 70% yield; mp 99-102 ^o^C

*Intermediate* i3: 4-(4-(hydroxy-3-methoxyphenyl)but-3-en-2-one

yellow solid, 69% yield; mp 125-128 ^o^C

*Intermediate* i4: 4-(4-(dimethylamino)phenyl)but-3-en-2-one

yellow solid, 77% yield; mp 123-125^o^C

*Intermediate* i5: 4-(4-methoxyphenyl)but-3-en-2-one

yellow solid, 74% yield; mp 68-70^o^C

**Preparation of the hydrazone derivatives**

In a reaction flask, 0.50 mmol of each intermediate (i1-i5), 0.471 g of hydralazine drug (previously pulverized with using mortar and pestle and containing 0.50 mmol drug equivalent), 5.0 ml of absolute ethanol, 4.0 ml of distilled water, 3.0 ml of glacial H_3_CCOOH and 3 drops of concentrated H_2_SO_4_ were mixed. The reaction mixture was closed and left to react at room temperature for 6 h with Constant magnetics tirring. The resulting mixture was filtered and the filtrate collected in a beaker. The residue retained on the paper was discarded (excipients). To the filtrate was added 20 ml of an ice cold aqueous solution of 5.0% (w/v) NaHCO_3_. The solid formed was filtered, washed with distilled water and dried at room temperature (protected from light). The products were purified by chromatographic column, using silica gel as stationary phase, and the mixture of hexane:ethylacetate (8:2 v/v) as eluent. The hydrazone derivatives (H1-H5) were characterized by melting point determination, FTIR, ^1^H-NMR and ^13^C-NMR. These purified products were used *in vivo* tests to assess antinociceptive and anti-inflammatory activities.

**Hydrazone derivatives properties**

Hydrazone H1 2-(3-(2-(phthalazin-1-yl)hydrazono)but-1-en-1-yl)phenol: orange solid, 46% yield, mp 263-265 ^o^C. FTIR (KBr, cm^-1^): 3107 (ʋN–H), 1605 (ʋC=N azomethine), 1584 and 1458 (ʋC=C). ^1^H-NMR (400 MHz, DMSO-d_6_, δppm): 2.30 (s, 3H, CH_3_); 6.85 (m, 2H, Ar-H); 7.12 (m, 2H, =CH + Ar-H); 7.27 (dd, 1 H, =CH, J = 16.63 Hz); 7.48 (d, 1H, Ar-H); 7.70 (m, 3H, Ar-H); 8.02 (s, 1H, Ar-H); 8.30 (d, 1H, Ar-H); 9.88 (s, 1H, OH) e 11.72 (s, 1H, NH). ^13^C-NMR (100 MHz, DMSO-d_6_, δ ppm): 12.47; 116.37; 119.85; 123.84; 124.17; 126.78; 126.82; 127.15; 127.42; 128.27; 129.62; 130.68; 132.14; 132.51; 137.90; 146.77; 155.61 e 159.87 (C=N, azomethine).

Hydrazone H2 4-(3-(2-(phthalazin-1-yl)hydrazono)but-1-en-1-yl)phenol: yellow solid, 44% yield, mp 240-242 ^o^C. FTIR (KBr, cm^-1^): 3243 (ʋN–H), 1605 (ʋC=N azomethine), 1584 and 1512 (ʋC=C). ^1^H-NMR (400 MHz, DMSO-d_6_, δ ppm): 2.28 (s, 3H, CH_3_); 6.78 (d, 2H, Ar-H); 6.93 (d, 1H, =CH, J = 16.50 Hz); 7.03 (d, 1H, =CH, J = 16.50 Hz); 7.40 (d, 2H, Ar-H); 7.70 (m, 3H, Ar-H); 8.02 (s, 1H, Ar-H); 8.30 (d, 1H, Ar-H); 9.73 (s, 1H, OH) e 11.68 (s, 1H, NH). ^13^C-NMR (100 MHz, DMSO-d_6_, δ ppm): 12.28; 115.78 (2C); 123.76; 126.41; 126.84; 127.04; 127.64; 127.85; 128.24 (2C); 131.74; 132.08; 132.95; 137.44; 146.24; 157.86 e 159.30 (C=N, azomethine).

Hydrazone H3 2-methoxy-4-(3-(2-(phthalazin-1-yl)hydrazono)but-1-en-1-yl)phenol: yellow solid, 40% yield, mp 241-243 ^o^C. FTIR (KBr, cm^-1^): 3264 (ʋN–H), 1604 (ʋC=N azomethine), 1584 and 1511 (ʋC=C). ^1^H-NMR (400 MHz, DMSO-d_6_, δ ppm): 2.31 (s, 3H, CH_3_); 3.84 (s, 3H, OCH_3_); 6.80 (d, 1H, =CH, J = 8.04 Hz); 7.00 (m, 3H, =CH + Ar-H); 7.17 (d, 1H, Ar-H); 7.73 (m, 3H, Ar-H); 8.04 (s, 1H, Ar-H); 8.32 (m, 1H, Ar-H); 9.31 (s, 1H, OH) e 11.66 (s, 1H, NH). ^13^C-NMR (100 MHz, DMSO-d_6_, δ ppm): 12.17; 55.44; 109.64; 120.44; 123.58; 126.25; 126.67; 126.87; 127.70; 128.23; 131.58; 131.94; 133.10; 137.31; 146.12; 147.11; 147.75 e 159.08 (C=N, azomethine).

Hydrazone H4 *N*,*N*-dimethyl-4-(3-(2-(phthalazin-1-yl)hydrazono)but-1-en-1-yl)aniline: orange solid, 51% yield, mp 176-178 ^o^C. FTIR (KBr, cm^-1^): 3242 (ʋN–H), 1601 (ʋC=N azomethine), 1585 and 1519 (ʋC=C). ^1^H-NMR (400 MHz, DMSO-d_6_, δ ppm): 2.30 (s, 3H, CH_3_); 2.95 (s, 6H, N(CH_3_)_2_); 6.74 (d, 2H, Ar-H); 6.90 (d, 1H, =CH, J = 16.56 Hz); 7.02 (d, 1H, =CH, J = 16.56 Hz); 7.41 (d, 2H, Ar-H); 7.72 (m, 3H, Ar-H); 8.02 (s, 1H, Ar-H); 8.30 (m, 1 H, Ar-H) e 11.62 (s, 1H, NH). ^13^C-NMR (100 MHz, DMSO-d_6_, δ ppm): 12.09; 39.77; 112.11; 123.53; 124.37; 125.84; 126.21; 126.78; 126.85; 127.73; 131.54; 131.83; 133.19; 137.15; 145.81; 150.16; 155.40 e 159.32 (C=N, azomethine).

Hydrazone H5 1-(2-(4-(4-methoxyphenyl)but-3-en-2-ylidene)hydrazinyl)phthalazine: yellow solid, 48% yield, mp 165-168 ^o^C. FTIR (KBr, cm^-1^): 3254 (ʋN–H), 1606 (ʋC=N azomethine), 1588 and 1508 (ʋC=C). ^1^H-NMR (400 MHz, DMSO-d_6_, δ ppm): 2.30 (s, 3H, CH_3_); 2.95 (s, 6H, N(CH_3_)_2_); 6.74 (d, 2H, Ar-H); 6.90 (d, 1H, =CH, J = 16.56 Hz); 7.02 (d, 1H, =CH, J = 16.56 Hz); 7.41 (d, 2H, Ar-H); 7.72 (m, 3H, Ar-H); 8.02 (s, 1H, Ar-H); 8.30 (m, 1 H, Ar-H) e 11.62 (s, 1H, NH). ^13^C-NMR (100 MHz, DMSO-d_6_, δ ppm): 12.09; 39.77; 112.11; 123.53; 124.37; 125.84; 126.21; 126.78; 126.85; 127.73; 131.54; 131.83; 133.19; 137.15; 145.81; 150.16; 155.40 e 159.32 (C=N, azomethine).

**^1^H-NMR of the hydrazone derivatives**

*^1^H-NMR spectra (400 MHz) in DMSO-d6 of hydrazone H1*

*^1^H-NMR spectra (400 MHz) in DMSO-d6 of hydrazone H2*

*^1^H-NMR spectra (400 MHz) in DMSO-d6 of hydrazone H3*

*^1^H-NMR spectra (400 MHz) in DMSO-d6 of hydrazone H4*

*^1^H-NMR spectra (400 MHz) in DMSO-d6 of hydrazone H5*

**^13^C-NMR of the hydrazone derivatives**

*^13^C-NMR spectra (400 MHz) in DMSO-d6 of hydrazone H1*

*^13^C-NMR spectra (400 MHz) in DMSO-d6 of hydrazone H2*

*^13^C-NMR spectra (400 MHz) in DMSO-d6 of hydrazone H3*

*^13^C-NMR spectra (400 MHz) in DMSO-d6 of hydrazone H4*

*^13^C-NMR spectra (400 MHz) in DMSO-d6 of hydrazone H5*

**FTIR of the hydrazone derivatives**

*FTIR spectra (KBr) of hydrazone H1*


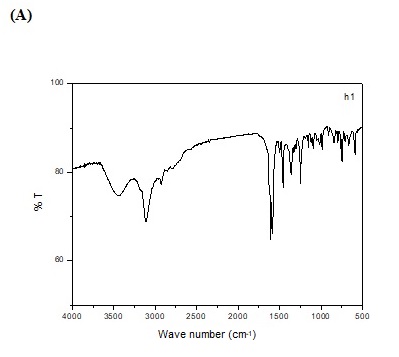


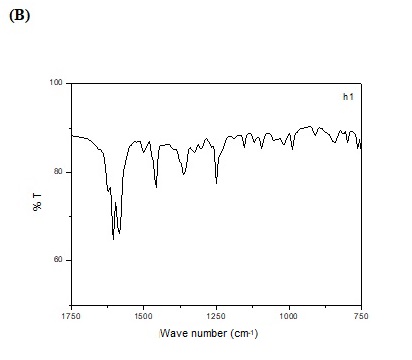


Peaks (cm^-1^):3447 (ʋO–H), 3107 (ʋN–H), 1605 (ʋC=N azomethine), 1584 e 1458 (ʋC=C), 1362 (ẟO–H), 1250, 1153 e 1093 (ʋC–O) e 1015 (ʋC–N).

*FTIR spectra (KBr) of hydrazone H2*

**
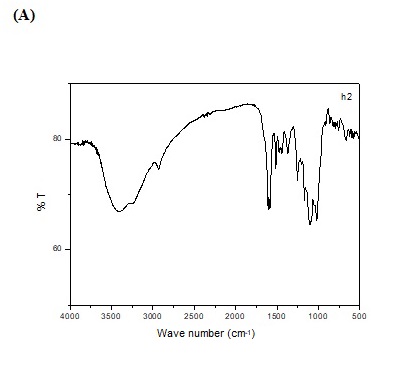
**

**
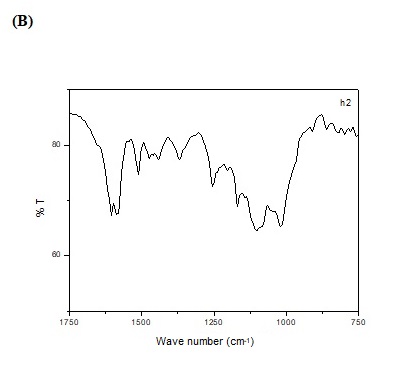
**

*Peaks(cm^-1^):3408 (ʋO–H), 3243 (ʋN–H), 1605 (ʋC=N azomethine), 1584 e 1512 (ʋC=C), 1368 (ẟO–H), 1254, 1168, 1102 (ʋC–O) e 1018 (ʋC–N).*

*FTIR spectra (KBr) of hydrazone H3*

**
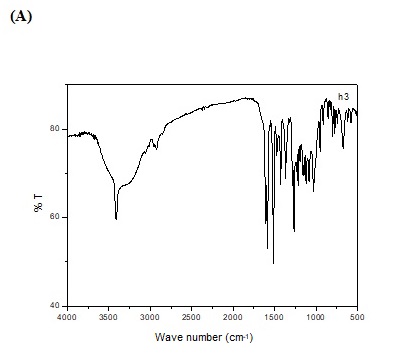
**

**
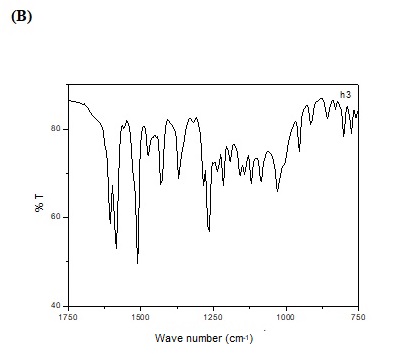
**

*Peaks (cm^-1^):3408 (ʋO–H), 3264 (ʋN–H), 2924 (ʋCH_3_eter), 1605 (ʋC=N azomethine), 1584 e 1511 (ʋC=C), 1431 (ẟCH_3_eter), 1370 (ẟO–H), 1265, 1215 e 1120 (ʋC–O–C), 1086 e 955 (ʋC–O) e 1029 (ʋC–N).*

*FTIR spectra (KBr) of hydrazone H4*

**
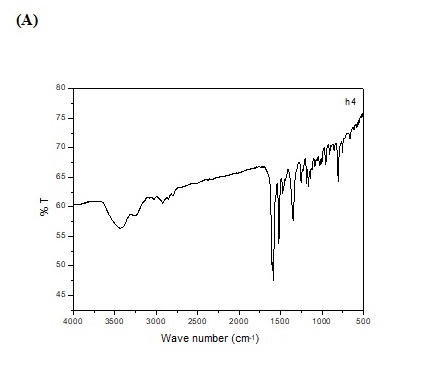
**

**
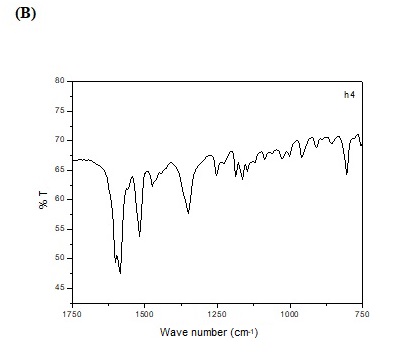
**

*Peaks (cm^-1^): 3242 (ʋN–H), 1601 (ʋC=N azomethine), 1585 e 1519 (ʋC=C), 1350 (ʋC–N) e 804 (ẟC–H).*

*FTIR spectra (KBr) of hydrazone H5*


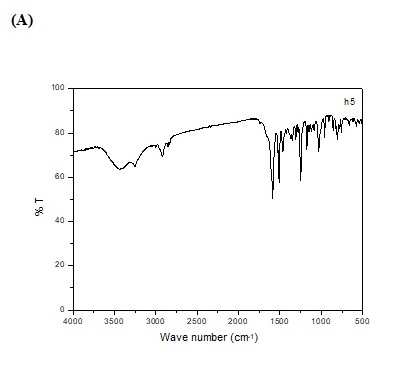


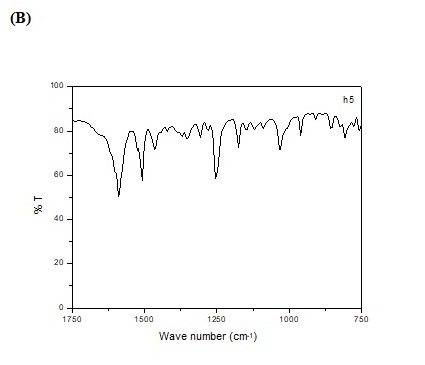


Peaks (cm^-1^):3254 (ʋN–H), 2921 (ʋCH_3_eter), 1605 (ʋC=N azomethine, “shoulder peak”), 1588, 1508 (ʋC=C), 1465 (ẟCH_3_eter), 1252 e 1177 (ʋC–O–C) e 1032 e 961 (ʋC–O).
