# Supplementary material for: Antinociceptive and anti-inflammatory effects of hydrazone derivatives and their possible mechanism of action in mice
Source: PLoS One. 2021 Nov 24;16(11):e0258094. doi: 10.1371/journal.pone.0258094 (PMC8612535; doi:10.1371/journal.pone.0258094)
Supplement: Attachment — Submitted filename: Response to Reviewers.docx [file pone.0258094.s003.docx]

**Response to Reviewers**

***Is the manuscript technically sound, and do the data support the conclusions?**

Yes.

*** Has the statistical analysis been performed appropriately and rigorously?**

All statistical analysis was performed using GraphPad Prism 5.0. Values are expressed as the mean ± SEM, where a indicates *p* < 0.05, significantly different from the control group, according to ANOVA, followed by Tukey's test.

***Have the authors made all data underlying the findings in their manuscript fully available?**

Yes.

***Is the manuscript presented in an intelligible fashion and written in standard English?**

Yes.

*** The rationale for choosing to make hydrazone derivatives and testing them in the animal models is not well described.**

The rationale for using hydrazine derivatives in animal models has been improved.

*** Page 5: animal weight 30 +/- 40 g and humidity 60 +/- 80% must be wrong.**

- All experiments were conducted using 8-week-old male Swiss mice (Mus musculus) (30-40 g).

According to the FCF-IQ/USP Production and Experimentation Laboratory Animal Care and Procedures Manual, the average weight of the adult male mouse (8 weeks) is about 40 g.

Several articles carried out antinociceptive tests with mice in this weight range:

- 30-35 g (Silva et al., 2018);
- 25-35 g (Macêdo, et al., 2020);
- 25-35 g (Hort et al., 2018).

- There was a typo regarding humidity conditions, we have fixed it (60-80% instead of 60 ± 80%). This condition was also used in other articles (Silva et al., 2018; Alves et al., 2020).

***Please describe the rationale for choosing 20 and 40 mg/kg doses to test.**

We used Silva et al. (2018) article as a reference for the choice of doses tested.

*** The Artemia salina toxicity test needs a more detailed description.**

We have detailed this test as recommended.

*** Is the indomethacin and morphine data in figures 1-5 different for each group or is the same data represented multiple times. The latter would be unacceptable without clearly stating so.**

In figures 2-6 (results of the abdominal writhing test induced by acetic acid) the results of indomethacin and morphine were the same, as animals from the same group were used, exposed to the same conditions, and all tests were performed on the same day. Therefore, we did not see the need to repeat evaluations of positive controls (indomethacin and morphine).

*** Page 18: Proof needs to be provided for the statement that ‘H5 is more chemically stable’.**

Both have stability, we made a correction in the text. H4 and H5 are pharmacologically and chemically similar, so we use H5 to perform the other pharmacological tests. Also, H5 was available in greater quantities to proceed with pharmacological testing.

*** The mention of celecoxib and cox-2 on page 19 comes out of nowhere and is only brought up, but not discussed. Similarly on page 21, the mention of bergamot and flavonoids seem irrelevant.**

- Mention of celecoxib and cox-2 on page 19: We have included a brief discussion on this specific topic of the discussion.

- Mention of bergamot and flavonoids: To confirm the antinociceptive effect involving peripheral opioid receptors, we found in the scientific literature that the bergamot essential oil (BEO)-induced antinociception possibly occurred through μ-opioid and κ-opioid receptors in the periphery. Beyond that, the capacity of flavonoids to decrease hyperalgesia can also involve µ and δ-opioid receptors.

*** The rationale for the use of each pharmacological inhibitor is poorly described. For example why was the ondansetron used.**

We conducted tests with several pharmacological blockers to investigate the possible mechanism of action of H5 through known signaling pathways, as much as possible. The pharmacological blockers used were:

- L-NAME: to reverse the involvement of nitric oxide. As described in the submitted article, a sufficient increase of NO concentration boosts the cGMP production and leads to the activation of glutamatergic receptors, which make the NO concentrations directly associated with nociception (Kawano et al, 2009; Sachs et al., 2004; Peana et al., 2006; Vale et al., 2007; Próspero et al., 2018).

- Naloxone: to investigate the involvement of opioid receptors, which are directly involved in central and peripheral pain. Similar Silva et al. (2018).

- Ondansetron: was used to investigate the involvement of serotonergic receptors in the nociception signaling pathway. Diverse serotonin (5-HT) receptors are present in the central and peripheral nervous systems involved in nociception (Kayser et al., 2010; Vujović et al., 2019).

- Atropine: was used to investigate the involvement of muscarinic receptors in the antinociceptive effect of H5.

- Glibenclamide: was used to investigate the involvement of the ATP-sensitive potassium channels in the antinociceptive effect of H5.

- Ruthenium red: was used to investigate the involvement of the vanilloid system in the antinociceptive effect of H5.

*** Given that L-Arg decreases licking time more than H5+ L-Arg, means that no conclusion can be drawn about any relation to L-Arg and H5. Any such conclusion needs to be removed (e.g. on page 22).**

We have agreed with the reviewer and proceeded with modifications.

*** The statement “shows that H5 (20 mg/kg, p.o.) significantly reduced (p<0.05) histamine induced paw edema at 30, 60, 90, 120, and 150 minutes, suggesting the involvement of histamine receptors in its anti-inflammatory effect” is too strong and needs to be in line with what the data really allows for.**

The change was done in the text.

*** Why was the rotarod test performed, one would expect H5 to be compared to a analgesic or anti-inflammatory drug that does perform poorly in this test.**

The intention of carrying out the route-rod test was only to assess the influence on motor coordination, which was found.

*** What were the positive and negative controls for the Artemia salina test.**

The positive control was paracetamol and the negative control was saline.

*** Page 31 The sentence “…the substance that has an LC50 value less than 1000 μg/ml compared to Artemia salina.” does not make sense.**

According to the literature, the cytotoxic activity against A. salina was considered weak when the LC50 was between 500 and 1000 μg/ml, moderate when the LC50 was between 100 and 500 μg/ml, as strong when the LC50 ranged from 0 to 100 μg/ml. In this sense, the results indicated that H5 present moderate toxicity after 24 hours and strong toxicity after 48 hours since it presented LC50 of 210.6 µg/ml and 81.95 µg/ml, respectively (Table 2).

*** Page 32: For validation, redocking studies were performed, comes out of nowhere. Validation of what. Re-docking of what. Why is this molecule docked at COX-2.**

- For validation of the H5 molecule, H5 redocking studies were performed.

- We agree with the reviewer, the procedure is not clear. Any docking studies need to be validated by re-docking the ligand in complex with the X-ray crystallographic structure of a given target, to verify if the scoring function can find the best affinity between the ligand and the target. In this case, Meloxicam (MXM) was re-docked. Compound H5 was docked because is a candidate to be an antinociceptive and anti-inflammatory drug. It is known that pharmacological inhibition of COX-2 relieves symptoms of inflammation and pain.

All the corrections were done in the text.

*** Meloxicam is used in the docking, but is not used at all in the in vivo models to make a more logical comparison.**

Meloxicam and indomethacin are NSAIDs, as both act by inhibiting COX-2 and promoting an anti-nociceptive and anti-inflammatory effect. For *in vivo* models the available drug was indomethacin, so we used this molecule as a positive control.

*** Page 33: “This may explain the H5 markable antinociceptive effect.” Is way too ambitious of a statement.**

The correction was done in the text.

*** Figure 22 and 23 the angle of view provides inferior insight into the binding mode/site.**

We improved the visualization of the H5 docking figure. Now, we think we have a better angle. The 2D figure was shown to give more insight into the binding site mode.

***Figures need to be pooled into multi-panel figures to reduce the figure number down from 23 to a more manageable number.**

All the corrections were done in the manuscript.

*** There are numerous grammatical errors and instances of dubious word choices in the manuscript, too many to list, but it will be valuable to re-read the manuscript and improve where possible. Purely as example: sentence 1 of the abstract “pain and inflammation ..resulting from imminent tissue damage”. Sentence 3 of the abstract starts with “therefore”, but is not really a logical continuation of the prior sentence. Sentence 6 ”greatest potential” should read “greatest potency”.**

All the corrections were done in the text.

*** Instead of stating “ at the highest doses tested” rephrase as “both tested doses”.**

All the corrections were done in the text.

*** What is the reason for administering reference drugs 30 minutes prior to nociceptive agent but test drugs being administered at 1 hour prior to nociceptive agent? Any rationale for this difference in time intervals?**

The drugs used as positive controls and antagonists were administered intraperitoneally (i.p.) therefore, their effect has a faster onset. Because of that, the literature indicates administration 30 min before the nociceptive agent. Hydrazones, on the other hand, were administered orally (p.o.) so their absorption takes a little longer. In this case, the literature indicates administration 1h before the nociceptive agent. We used the work of Silva et al. (2018) as a reference work.

*** Each graph is placed separately as a figure. This makes the manuscript unnecessarily bulky and long. Could the authors please consolidate their findings? For example, Figure 2 for acetic acid test can comprise of graphs for H1 through H5 put together (current figures 2-6) and so on. Also, there are a massive number of supplementary figures, which could again be consolidated.**

All the corrections were done in the text.

*** It would be helpful for the authors to insert a table listing all statistical analyses performed with their respective exact p-values. Right now, significance is only indicated as p<0.05 in figure legends.**

Table p values for each data group analyzed (p<0.05).

| **Figure** | **Overall p value (ANOVA)** | **p value (post-test comparison)** |
| --- | --- | --- |
| Fig. 2A | 0.000000040609 | 0.00000053922(Control vs. H1 20 mg/kg) |
|  |  | 0.000000081718(Control vs. H1 40 mg/kg) |
|  |  | 0.000000014611(Control vs. Indomethacin) |
|  |  | 0.000000038021 (Control vs. Morphine) |
| Fig. 2B | 0.000000000080570 | 0.000000014106 (Control vs. H2 20 mg/kg) |
|  |  | 0.000000055374(Control vs. H2 40 mg/kg) |
|  |  | 0.00000003486 (Control vs. Indomethacin) |
|  |  | 0.00000005861 (Control vs. Morphine) |
| Fig. 2C | 0.0000096297 | 0.00079477(Control vs. H2 20 mg/kg) |
|  |  | 0.00046048(Control vs. H2 40 mg/kg) |
|  |  | 0.000064232 (Control vs. Indomethacin) |
|  |  | 0.000015961 (Control vs. Morphine) |
| Fig. 2D | 0.0000000059572 | 0.00000085659 (Control vs. H2 20 mg/kg) |
|  |  | 0.000000035286(Control vs. H2 40 mg/kg) |
|  |  | 0.000000098075 (Control vs. Indomethacin) |
|  |  | 0.000000013041 (Control vs. Morphine) |
| Fig. 2E | 0.012084 | 0.044104 (Control vs. H2 20 mg/kg) |
|  |  | 0.044140(Control vs. H2 40 mg/kg) |
|  |  | 0.01398 (Control vs. Indomethacin) |
|  |  | 0.02604 (Control vs. Morphine) |
| Fig. 3 A | 0.0080451 | 0.0039525 (Control vs. Morphine) |
| Fig. 4A | 0.00060544 | 0.00090216(Control vs. H1 20 mg/kg) |
|  |  | 0.0092175 (Control vs. H1 40 mg/kg) |
|  |  | 0.00000024934 (Control vs. Indomethacin) |
|  |  | 0.00000040579 (Control vs. Morphine) |
|  |  | 0.00014615 (Indomethacin vs. H1 40 mg/kg) |
|  |  | 0.0025929 (Morphine vs. H1 40 mg/kg) |
| Fig. 3B | 0.0080451 | 0.0021747 (Control vs. Morphine) |
| Fig. 4 B | 0.000027793 | 0.041411 (Control vs. H2 20 mg/kg) |
|  |  | 0.00057994 (Control vs. Indomethacin) |
|  |  | 0.00083145 (Control vs. Morphine) |
|  |  | 0.0017733 (Indomethacin vs. H2 40 mg/kg) |
|  |  | 0.0025487 (Morphine vs. H2 40 mg/kg) |
| Fig. 3C | 0.032120 | 0.016980 (Control vs. Morphine) |
| Fig. 4C | 0.000000087205 | 0.010603 (Control vs. H3 20 mg/kg) |
|  |  | 0.00043114 (Control vs. H3 40 mg/kg) |
|  |  | 0.00000036463 (Control vs. Indomethacin) |
|  |  | 0.00000059070 (Control vs. Morphine) |
| Fig. 3D | 0.023847 | 0.0094831 (Control vs. Morphine) |
| Fig. 4D | 0.00000000083947 | 0.0000000085625 (Control vs. H4 20 mg/kg) |
|  |  | 0.00000054353 (Control vs. H4 40 mg/kg) |
|  |  | 0.0000000071595 (Control vs. Indomethacin) |
|  |  | 0.000000012084 (Control vs. Morphine) |
| Fig. 3E | 0.0091879 | 0.014445 (Control vs. Morphine) |
| Fig. 4E | 0.000000000034939 | 0.000000034360 (Control vs. H5 20 mg/kg) |
|  |  | 0.0000000024329 (Control vs. H5 40 mg/kg) |
|  |  | 0.0000000044419 (Control vs. Indomethacin) |
|  |  | 0.0000000076611(Control vs. Morphine) |
| Fig. 5 A | 0.00043935 | 0.00072346 (Control vs. Morphine) |
| Fig. 5 B | 0.00000040120 | 0.000094298 (Control vs. H5 20 mg/kg) |
|  |  | 0.0000043385 (Control vs. Morphine) |
|  |  | 0.000037474 (Morphine vs. Naloxone) |
|  |  | 0.00091947 (H5 20 mg/kg vs. Naloxone) |
| Fig. 6 B | 0.00014799 | 0.0066284 (Control vs. L-Arg) |
|  |  | 0.00022349 (Control vs. H5 20 mg/kg) |
|  |  | 0.022578 (L-NAME vs. H5 20 mg/kg) |
|  |  | 0.0074382 (L-NAME vs. L-Arg) |
| Fig. 7 B | 0.0043011 | 0.0083922 (Control vs. H5 20 mg/kg) |
|  |  | 0.013164 (Control vs. Ondansetron+H5) |
|  |  | 0.0014361 (Ondansetron vs. H5 20 mg/kg) |
|  |  | 0.022664 (Ondansetron vs. Ondansetron+H5) |
| Fig. 8 B | 0.0000016054 | 0.000012141 (Control vs. Atropine)  0.000034765 (Control vs. H5 20 mg/kg) |
|  |  | 0.0000062806 (Control vs. Atropine+H5) |
| Fig. 9 B | 0.0014055 | 0.0044870 (Control vs.Glibenclamide) |
|  |  | 0.0042503 (Control vs. H5 20 mg/kg) |
|  |  | 0.014696 (Control vs. Glibenclamide+H5) |
| Fig. 10 B | 0.0015330 | 0.0013539 (Control vs. H5 20 mg/kg) |
|  |  | 0.015086 (Control vs. H5+VR) |
| Fig. 11 | 0.0054614 | 0.023577 (Control vs. H5 20 mg/kg) |
|  |  | 0.033974 (Control vs. H5 40 mg/kg) |
|  |  | 0.0049736 (Control vs. Dexamethasone) |
| Fig. 12 | 0.0011408 | 0.00040408 (Control vs. Saline) - 1 h |
|  |  | 0.015529 (Control vs. H5 20 mg/kg) - 1 h |
|  |  | 0.029918 (Control vs. H5 40 mg/kg) - 1 h |
|  |  | 0.025507 (Control vs. Indomethacin) - 1 h |
|  | 0.0014251 | 0.0012353 (Control vs. Saline) - 2 h |
|  |  | 0.027574 (Control vs. H5 20 mg/kg) - 2 h |
|  |  | 0.015879 (Control vs. H5 40 mg/kg) - 2 h |
|  |  | 0.0052421 (Control vs. Indomethacin) - 2 h |
|  | 0.00060088 | 0.00065564 (Control vs. Saline) - 3 h |
|  |  | 0.014908 (Control vs. H5 20 mg/kg) - 3 h |
|  |  | 0.021484 (Control vs. H5 40 mg/kg) - 3 h |
|  |  | 0.0024633 (Control vs. Indomethacin) - 3 h |
|  | 0.0000054690 | 0.0000033275 (Control vs. Saline) - 4 h |
|  |  | 0.00054564 (Control vs. H5 20 mg/kg) - 4 h |
|  |  | 0.0013666 (Control vs. H5 40 mg/kg) - 4 h |
|  |  | 0.00013699 (Control vs. Indomethacin) - 4 h |
| Fig. 13 | 0.00003863 | 0.000088694(Control vs. H5 2m mg/kg) - 30 min |
|  |  | 0.00022278(Control vs. Indomethacin) - 30 min |
|  | 0.00033049 | 0.0011142(Control vs. H5 2m mg/kg) - 60 min |
|  |  | 0.00096915(Control vs. Indomethacin) - 60 min |
|  | 0.00025008 | 0.00079882(Control vs. H5 2m mg/kg) - 90 min |
|  |  | 0.00079882(Control vs. Indomethacin) – 90 min |
|  | 0.00050503 | 0.0016219(Control vs. H5 2m mg/kg) - 120 min |
|  |  | 0.0014738(Control vs. Indomethacin) - 120 min |
|  | 0.0046013 | 0.0063718 (Control vs. H5 2m mg/kg) - 150 min |
|  |  | 0.027370 (Control vs. Indomethacin) - 150 min |
| Fig. 14 | 0.0415 | 0.9575 (Control vs. H5 20 mg/kg) – 0.5 h |
|  |  | 0.98141 (Control vs. H5 40 mg/kg) – 0.5 h |
|  |  | 0.047355 (Control vs. Diazepam) – 0.5 h |
|  | 0.00843 | 0.006836 (Control vs. H5 20 mg/kg) – 1.0 h |
|  |  | 0.04771 (Control vs. H5 40 mg/kg) – 1.0 h |
|  |  | 0.0063169 (Control vs. Diazepam) – 1.0 h |
|  | 0.001407 | 0.006471 (Control vs. H5 20 mg/kg) – 1.5 h |
|  |  | 0.0054422 (Control vs. H5 40 mg/kg)– 1.5 h |
|  |  | 0.001947 (Control vs. Diazepam) – 1.5 h |
|  | 0.006393 | 0.013828 (Control vs. H5 20 mg/kg) – 2.0 h |
|  |  | 0.042308 (Control vs. H5 40 mg/kg)– 2.0 h |
|  |  | 0.010538 (Control vs. Diazepam) – 2.0 h |

*** It is unclear if an unbiased video recording and analysis software was used to document pain behavior after administering the drugs. If behaviors were recorded manually, what was done to avoid experimenter error and bias?**

To minimize errors, a double-blind study was carried out, that is, the experimenter who administered the drugs is different from the experimenter who performed the observation of the experiment, who did not know the substances tested on the animals he evaluated. The same experimenters performed the entire study, to avoid different variants or interpretations.

*** The authors did a good job of explaining the rationale and action of already established anti-nociceptive drugs used as controls. However, they missed explanations for specific actions for a few of these namely, Ondansetron, atropine, glibenclamide, etc.**

We conducted tests with several pharmacological blockers to investigate the possible mechanism of action of H5 through known signaling pathways, as much as possible. The pharmacological blockers used were:

- L-NAME: to reverse the involvement of nitric oxide. As described in the submitted article, a sufficient increase of NO concentration boosts the cGMP production and leads to the activation of glutamatergic receptors, which make the NO concentrations directly associated with nociception (Kawano et al, 2009; Sachs et al., 2004; Peana et al., 2006; Vale et al., 2007; Próspero et al., 2018).

- Naloxone: to investigate the involvement of opioid receptors, which are directly involved in central and peripheral pain. Similar Silva et al. (2018).

- Ondansetron: was used to investigate the involvement of serotonergic receptors in the nociception signaling pathway. Diverse serotonin (5-HT) receptors are present in the central and peripheral nervous systems involved in nociception (Kayser et al., 2010; Vujović et al., 2019).

- Atropine: was used to investigate the involvement of muscarinic receptors in the antinociceptive effect of H5.

- Glibenclamide: was used to investigate the involvement of the ATP-sensitive potassium channels in the antinociceptive effect of H5.

- Ruthenium red: was used to investigate the involvement of the vanilloid system in the antinociceptive effect of H5.

References:

Silva JC, Oliveira-Júnior RG, Silva MG, Lavor EM, Soares JMD, Saraiva SRGL, et al. LASSBio-1586, an *N*-acylhydrazone derivative, attenuates nociceptive behavior and the inflammatory response in mice. PLoS One. 2018; 13: e0199009. 9.

Macêdo LARO, Pacheco AGM, Saraiva SRGL, Silva JC, Oliveira-Júnior RG, Souza GR, Lavor EM, Silva MG, Ferro JNS,Barreto E, Oliveira VR,Almeida JRGS. Fractions of *Selaginellaconvoluta* (Arn.) Spring (Selaginellaceae) attenuate the nociceptive behavior events in mice. Brazilian Journal of Biology. 2020; 80: 57-65.

Hort MA, Silva-Júnior FMR, Garcia EM, Peraza GG, Soares A, Lerner C, Baisch ALM. Antinociceptive and Anti-inflammatory Activities of Marine Sponges *AplysinaCaissara*, *Haliclona*sp. and *DragmacidonReticulatum*. 2018; 61: e18180104.

Alves JNC, Sousa-Junior FAC, Stutz ETG, Silva TC. Effects of tea consumption of "french chives" (*allium schoenopranoprasumL*.) In mice submitted to a high-fat diet. Brazilian Journal of Development. 2020; 6: 73892-73903.

Kawano T, Zoga V, Callum JBM, Wu HE, Gemes G, Liang MY, et al. ATP-sensitive potassium currents in rat primary afferent neurons: biophysical, pharmacological properties, and alterations by painful nerve injury. Neuroscience. 2009; 162: 431–443.

Sachs D, Cunha FQ, Ferreira SH. Peripheral analgesic blockade of hypernociception: Activation of arginine/NO/cGMP/protein kinase G/ATP-sensitive K+ channel pathway. ProcNatlAcadSci U S A. 2004; 101: 3680–3685.

Peana AT, Marzocco S, Popolo A, Pinto A. (-)-Linalool inhibits in vitro NO formation: Probable involvement in the antinociceptive activity of this monoterpene compound. Life Sci. 2006; 78: 719–723.

Vale ML, Rolim DE, Cavalcante IF, Ribeiro RA, Souza MHLP. Role of NO/cGMP/KATP pathway in antinociceptive effect of sildenafil in zymosan writhing response in mice. Inflamm Res. 2007; 56: 83–88.

Próspero DFA, Reis-Filho AC, Piauilino CA, Lopes EM, Sousa DP, Almeida FRC. Effects of isopulegol in acute nociception in mice: Possible involvement of muscarinic receptors, opioid system and L-arginine/NO/cGMP pathway. ChemBiol Interact. 2018; 293: 55–60.

Kayser V, Bourgoin S, Viguier F, Michot B, Hamon M. Toward deciphering the respective roles of multiple 5-HT receptors in the complex serotonin-mediated control of pain. Pharmacolpain. 2010; 185–207.

Vujović KS, Vučković S, Vasović D, Medić B, Stojanović R, Divac N, et al. Involvement of serotonergic and opioidergic systems in the antinociceptive effect of ketamine-magnesium sulphate combination in formalin test in rats. PharmacolReports. 2019; 71: 1014–1019.
