# Supplementary material for: Antinociceptive and anti-inflammatory effects of hydrazone derivatives and their possible mechanism of action in mice
Source: PLoS One. 2021 Nov 24;16(11):e0258094. doi: 10.1371/journal.pone.0258094 (PMC8612535; doi:10.1371/journal.pone.0258094)
Supplement: Attachment — Submitted filename: Response to Reviewers.docx [file pone.0258094.s004.docx]

**Response to Reviewers**

*** The authors addressed several of my prior concerns, but several were not addressed. It is critical that in figures 2-4 it Is made clear that the indomethacin and morphine data are identical in each panel.**

**Major/critical concerns:**

**Figure 2-4: You have to explicitly state that in each panel the indomethacin and morphine are identical/copy pasted. Not doing so gives the false and unethical impression that you ran a new positive control each time.**

Thank you for your observations. The positive controls used in the graphs of figure 2 were the same, as the experiments were carried out on the same day, the animals used had the same age and weight range, in addition to being exposed to the same environmental conditions.

The same occurred for figures 3 and 4.

*** The statement “The anti-inflammatory effect of H5 appears to involve histaminergic receptors” is not based on the data. Just because the molecule can reduce inflammation induced by histamine does not mean it acts through histaminergic receptors. As a simple analogy: If morphine reduces pain from gunshot wounds, it doesn’t mean morphine acts on guns.**

Thank you for this consideration. I guess we might have a semantic problem in the referred sentence. Perhaps the word “appears” sounded more like a certainty than a possibility, but it is not what we meant. In this sense, the text has been improved to fix misinterpretations about what we judge as possible participation of histaminergic receptors in the anti-inflammatory effect of H5.

*** It seems that H5 is more toxic than the positive control, please explain.**

Regarding Table 1, a similar result of H5 was observed in relation to the positive control at higher concentrations. The results indicated that H5 present moderate toxicity after 24 hours and strong toxicity after 48 hours since it presented LC50 of 210.6 µg/ml and 81.95 µg/ml, respectively (Table 2).

*** Please remove that pain results from imminent tissue damage from the 1st sentence of the abstract and introduction.**

We have proceeded with this modification. Thank you.

*** The sentence “Such panorama indicates that the application of classical in vivo animal experiments has an important role in drug discovery” is not very clear.**

Thank you for this consideration. We have agreed with the reviewer and proceeded with some modifications.

*** What is the point of the statement “Additionally, a variety of hydrazone derivatives have been developed to minimize gastrointestinal discomfort and toxicity, especially when it comes to analgesic drugs” minimizing GI discomfort and toxicity doesn’t really link to the current study where the antinociceptive and anti-inflammatory of the derivatives are being assessed.**

Thank you for your comment. GI discomfort and toxicity are disadvantages that greatly limit the use of analgesics and anti-inflammatory drugs. This phrase was added to emphasize an advantage of hydrazones over classical anti-inflammatories. However, it deserves some improvement.

*** Page 15 “When animals were pretreated with naloxone (1.5 mg/kg, i.p.), the pharmacological effect of H5 (20 mg/kg, p.o.) was completely reversed in the second phase of the test (Fig 5), suggesting that its peripheral antinociceptive response was involved at least in part with the opioid system.” Is repeated on page 16-17 “When animals were pretreated with naloxone, the pharmacological effect of H5 was completely reversed in the second phase of the formalin test, suggesting that its peripheral antinociceptive response is involved, at least in part, with the opioid system”.**

Thank you for this observation. We have proceeded with the correction.

*** Figure 15 the two interaction map is low resolution and hard to read. The docked molecule is very planar and the angle of the docked ligand hides a lot of the molecule, a small tilt would show much more of the ligand.**

We apologize for that. A high-resolution image replaced figure 15.

***** **For figures, please use individual dots for each datapoint instead of bar graphs.**

Thank you for this consideration. We have agreed with the reviewer and proceeded with some modifications. Figures 12, 13 and 14 were not self-explanatory in the requested model, so we left it in the previous model.
